# Are coagulation profiles in Andean highlanders with excessive erythrocytosis favouring hypercoagulability?

**DOI:** 10.1113/EP091670

**Published:** 2024-03-30

**Authors:** Benoit Champigneulle, François Caton, Landry Seyve, Émeric Stauffer, Aurélien Pichon, Julien V. Brugniaux, Michael Furian, Ivan Hancco, Blandine Deschamps, Lars Kaestner, Paul Robach, Philippe Connes, Pierre Bouzat, Benoit Polack, Raphael Marlu, Samuel Verges

**Affiliations:** ^1^ Univ. Grenoble Alpes, Inserm, CHU Grenoble Alpes, HP2 Grenoble France; ^2^ Department of Anaesthesia and Critical Care CHU Grenoble Alpes Grenoble France; ^3^ Univ. Grenoble Alpes, CNRS, LRP Grenoble France; ^4^ Hemostasis Laboratory Grenoble University Hospital Grenoble France; ^5^ Laboratoire Interuniversitaire de Biologie de la Motricité (LIBM) EA7424, Team ‘Vascular Biology and Red Blood Cell’ Université Claude Bernard Lyon 1, Université de Lyon Lyon France; ^6^ Laboratoire d'Excellence du Globule Rouge (Labex GR‐Ex), PRES Sorbonne Paris France; ^7^ Exploration Fonctionnelle Respiratoire, Médecine du Sport et de l'Activité Physique, Hospices Civils de Lyon, Hôpital Croix Rousse Lyon France; ^8^ Université de Poitiers, Laboratoire Move UR 20296, STAPS Poitiers France; ^9^ Dynamics of Fluids, Experimental Physics Saarland University Homburg Germany; ^10^ Theoretical Medicine and Biosciences, Medical Faculty Saarland University Homburg Germany; ^11^ National School for Mountain Sports, Site of the National School for Skiing and Mountaineering (ENSA) Chamonix France; ^12^ Univ. Grenoble Alpes, Inserm, CHU Grenoble Alpes, GIN Grenoble France; ^13^ Univ. Grenoble Alpes, CNRS, CHU Grenoble Alpes, TIMC‐IMAG Grenoble France

**Keywords:** blood coagulation, chronic mountain sickness, excessive erythrocytosis, hypoxia, thromboelastometry

## Abstract

Chronic mountain sickness is a maladaptive syndrome that affects individuals living permanently at high altitude and is characterized primarily by excessive erythrocytosis (EE). Recent results concerning the impact of EE in Andean highlanders on clotting and the possible promotion of hypercoagulability, which can lead to thrombosis, were contradictory. We assessed the coagulation profiles of Andeans highlanders with and without excessive erythrocytosis (EE+ and EE−). Blood samples were collected from 30 EE+ and 15 EE− in La Rinconada (Peru, 5100–5300 m a.s.l.), with special attention given to the sampling pre‐analytical variables. Rotational thromboelastometry tests were performed at both native and normalized (40%) haematocrit using autologous platelet‐poor plasma. Thrombin generation, dosages of clotting factors and inhibitors were measured in plasma samples. Data were compared between groups and with measurements performed at native haematocrit in 10 lowlanders (LL) at sea level. At native haematocrit, in all rotational thromboelastometry assays, EE+ exhibited hypocoagulable profiles (prolonged clotting time and weaker clot strength) compared with EE− and LL (all *P *< 0.01). At normalized haematocrit, clotting times were normalized in most individuals. Conversely, maximal clot firmness was normalized only in FIBTEM and not in EXTEM/INTEM assays, suggesting abnormal platelet activity. Thrombin generation, levels of plasma clotting factors and inhibitors, and standard coagulation assays were mostly normal in all groups. No highlanders reported a history of venous thromboembolism based on the dedicated survey. Collectively, these results indicate that EE+ do not present a hypercoagulable profile potentially favouring thrombosis.

## Abstract (highlights)

- **What is the central question of this study?**
  Are Andean highlanders with excessive erythrocytosis (EE+) exhibiting a hypercoagulable profile compared with highlanders without erythrocytosis (EE−) and lowlanders (LL)?
- **What is the main finding and its importance?**
  Despite normal plasma coagulation (thrombinography and levels of clotting factors and inhibitors), EE+ exhibited a hypocoagulable rotational thromboelastometry profile (prolonged clotting time and weaker clot strength) compared with EE− and LL. In EE+, haematocrit normalization at 40% corrected maximal clot firmness in rotational thromboelastometry FIBTEM tests, but not in EXTEM and INTEM tests, suggesting that platelets play a role in the native hypocoagulable profile.

## 1 INTRODUCTION

More than 80 million highlanders, living permanently in high‐altitude areas (i.e., >2500 m a.s.l.) worldwide, are chronically exposed to hypobaric hypoxia (Tremblay & Ainslie, 2021). High‐altitude natives, descendants of populations living at high altitude for thousands of years, have developed genetic adaptations that allow them to cope with their hypoxic environment (Azad et al., 2017; Pamenter et al., 2020). Different adaptive patterns have been described among the Andean and Tibetan populations, which represent the most studied high‐altitude populations (Beall, 2007; Moore, 2017). In particular, Andean populations exhibit higher haemoglobin concentration ([Hb]) than Tibetans (Mairbäurl et al., 2020) and are more susceptible to developing chronic mountain sickness (CMS) (Villafuerte & Corante, 2016; Villafuerte et al., 2022). Chronic mountain sickness is a maladaptive syndrome secondary to excessive erythrocytosis (EE), defined by an international consensus as a [Hb] of ≥21 g dL^−1^ in men and ≥19 g dL^−1^ in women (León‐Velarde et al., 2005) and including symptoms such as breathlessness, palpitations, sleep disturbance, cyanosis, dilatation of veins, paraesthesia, headache and tinnitus, although the exact pathophysiological mechanisms underlying EE and CMS symptoms remain unclear (Stauffer et al., 2020). It is estimated that 5%–10% of the worldwide highlander population suffers from EE and are therefore at risk of CMS, a prevalence that increases with the altitude of residence and with ageing and/or residency time at high altitude (Champigneulle et al., 2022; Villafuerte & Corante, 2016; Villafuerte et al., 2022). We previously reported a prevalence of EE of 44% in La Rinconada (Hancco, Bailly, et al., 2020), the highest city in the world (5100–5300 m a.s.l.), a gold‐mining city in southern Peru with an estimated population of ∼50,000 people (Champigneulle et al., 2024).

At sea level, other causes of polycythaemia, such as myeloproliferative diseases (polycythaemia vera), Chuvash polycythaemia and secondary polycythaemia, have been associated with an increased risk of thrombosis (Gordeuk et al., 2019; Nguyen et al., 2021), albeit this risk was not necessarily associated with increasing [Hb] (Gordeuk et al., 2019, 2020). Other factors, such as hypoxic exposure, have been associated with an increased thrombotic risk in polycythaemia vera (Zangari et al., 2013). However, higher haematocrit is known to promote venous and arterial thrombosis though several mechanisms, which can be slightly different according to low (veins) or high (arteries) shear rates (Weisel & Litvinov, 2019).

Despite being a potential thrombotic factor, the impact of EE on venous thrombosis risk has not been investigated in Andean highlanders. On the arterial side, it has been suggested that the increased haematocrit caused by altitude polycythaemia might increase the risk of cardiovascular events and strokes, although the frequent association of EE/CMS with common cardiovascular risk factors might be a confounding factor (Ortiz‐Prado et al., 2022; Villafuerte & Corante, 2016; Villafuerte et al., 2022). Furthermore, although the impact of acute altitude exposure on haemostasis in lowlanders (LL) ascending to high altitude has been studied extensively and reviewed recently (Treml et al., 2022), the impact of permanent residence at high altitude and the potential repercussions of EE on the haemostatic system remain poorly investigated (DeSouza et al., 2021; Hancco, Champigneulle, et al., 2020; Jiang et al., 2021; Wang et al., 2023; Yin et al., 2022). In particular, two recent studies on Andean highlanders reported possibly conflicting results. An investigation of the coagulation–fibrinolytic axis conducted in Cerro de Pasco (∼4300 m a.s.l., Peru) did not support a hypercoagulable pattern in highlanders with EE (noted hereafter as EE+) (DeSouza et al., 2021), and we previously reported, in a preliminary study conducted in La Rinconada (∼1000 m higher), lower bleeding and clotting times in EE+ compared with those highlanders without EE (noted hereafter as EE−) (Hancco, Champigneulle, et al., 2020). Additionally, only one of these previous studies comparing haemostatic status in EE+ and EE− highlanders included a sea‐level control group, in order to highlight the potential effect of long‐term hypoxic exposure per se (Jiang et al., 2021). The lack of studies aimed at investigating the impact of EE might be explained, at least in part, by the difficulty of conduct complex biological assays in high‐altitude remote areas, offering limited research facilities (Hancco, Champigneulle et al., 2020). In a constrained environment, such as La Rinconada, haemostatic point‐of‐care devices, mainly thromboelastic assays, offer the possibility to assess the global dynamics of whole‐blood clotting in the field (Volod et al., 2022). Thereby, rotational thromboelastometry (ROTEM) and thromboelastography have been used successfully in the field, up to 5300 m a.s.l., to assess clot kinetics in LL acutely exposed to high altitude (Martin et al., 2017; Rocke et al., 2018), but never in healthy highlanders (i.e., those well‐adapted to high altitude, EE−) or those suffering from maladaptation (i.e., EE+).

Based on the findings from our preliminary study (Hancco, Champigneulle, et al., 2020), we hypothesized that EE+ Andeans might present a hypercoagulable state compared with EE− and LL. To test this hypothesis, we performed a field study in La Rinconada (5100–5300 m a.s.l.), assaying whole‐blood coagulation using ROTEM at both native and normalized haematocrits. Particular attention was paid on pre‐analytical sampling variables, in particular the citrate‐to‐blood ratio. In order to examine the coagulation cascade in Andeans highlanders, we completed this whole‐blood coagulation testing by measurements of clotting factor and inhibitor levels and by a thrombin generation assay.

## 2 MATERIALS AND METHODS

### 2.1 Ethical approval and study overview

To investigate both the effect of polycythaemia in maladapted highlanders suffering from EE and the potential effect of chronic hypoxic exposure in apparently well‐adapted highlanders on coagulation, we conducted a cross‐sectional study within the Expedition5300 research programme (Champigneulle et al., 2024). This study was approved by the ethics committee of the Universidad Peruana Cayetano Heredia (Lima, Peru, IRB number: 00003251) and conducted in accordance with the standards set by the *Declaration of Helsinki*, except for registration in a database. All participants were fully informed of the study in their native language and signed a written informed consent form before inclusion.

### 2.2 Experimental design

#### 2.2.1 Highlander participants

Haemostasis testing was performed on 45 male highlanders who were residents of La Rinconada (5100–5300 m a.s.l., Peru) for >3 years (median [25th–75th percentiles] residency time, 15 [7–20] years) and natives from the altiplano area (i.e., >3500 m a.s.l.). Highlander participants were recruited among the residents of La Rinconada who reported voluntarily to our temporary medical research laboratory located in the mining cooperative building. None of the highlander individuals was receiving anticoagulant or anticoagulant therapy, and none had a medical history of cardiovascular, metabolic or respiratory diseases that could induce secondary polycythaemia or interfere with the diagnosis of EE and CMS (León‐Velarde et al., 2005). None of the participants reported excessive alcohol consumption (i.e., more than two units per day). All of them were professionally active and mainly involved in mining activities (33 of 45, 73%).

#### 2.2.2 Lowlander participants

The same haemostasis testing was performed at a low altitude (210 m a.s.l.; Grenoble, France) on a sea‐level control group consisting of 10 healthy Caucasian LL without any high‐altitude sojourn in the previous months (≥2500 m a.s.l.), all of them being members of the Expedition5300 research team. None of the LL participants had a significant medical history (including known prothrombotic disease or bleeding diathesis), and they had not taken any chronic medications (except low‐dose combined oestrogen–progestin oral contraceptive for one LL female).

### 2.3 Clinical assessment of highlanders

Demographic and clinical data were recorded during an initial medical consultation. Highlanders were categorized as EE+ (*n* = 30) or EE− (*n* = 15), according to the [Hb] conventional threshold of 21 g dL^−1^ (León‐Velarde et al., 2005). A diagnosis of CMS was made in the case of a Qinghai CMS score ≥6, including the presence of EE. This score is based on the scoring of seven clinical signs or symptoms (breathlessness and/or palpitations, sleep disturbance, cyanosis, venous dilatation, paraesthesia, headache and tinnitus) from zero (absence of symptom) to three (severe symptom) and the absence or presence of EE (adding three points to the symptom subscore). The severity of CMS is then defined as mild (CMS score = 6–10), moderate (CMS score = 11–14) or severe (CMS score ≥ 15) (León‐Velarde et al., 2005). A validated questionnaire, translated into Spanish, was used to assess any history of venous thromboembolism (Frezzato et al., 1996).

### 2.4 Blood sampling: collection, citrate adaptation to haematocrit and plasma storage

All blood samples were collected through a venous antecubital puncture performed while the participants were at rest in supine position, using a butterfly system. Three millilitre Vacutainer tubes (Becton‐Dickinson, Le Pont‐de‐Claix, France), containing either 0.109 M (3.2%) trisodium citrate (1/10 volume) for the haemostasis assays or EDTA for complete blood count, were used.

The previously reported median haematocrit in highlanders from La Rinconada was ∼73% (Oberholzer et al., 2020), which is much higher than the accepted upper limit of 55% for standard citrated tubes (Kitchen et al., 2021). Specially, an increase in haematocrit from 50% to 75% corresponds to a halving of the plasma volume, resulting in a doubling of the citrate concentration in plasma. Therefore, the volume of citrate present in Vacutainer tubes must be corrected to ensure that haemostasis results are not affected by this artefact. The International Council for Standardization in Haematology recommends using the following formula to determine the appropriate citrate volume: *C* = 0.00185 × (100 − haematocrit) × *V*, where *C* is the volume of trisodium citrate (in millilitres) and *V* is the amount of blood (in millilitres) (Kitchen et al., 2021). This correction obviously requires obtaining haematocrit values before the sampling. Therefore, the haematocrit value was obtained from a capillary blood sample using the microcentrifuge method during the inclusion visit, immediately before venous blood sampling. Given that the sampling was performed in a temporary, in‐the‐field medical research laboratory installed in a challenging environment and considering the relatively small volumes of citrate to be removed, the procedure had to be simplified and optimized as follows. The quantity of trisodium citrate contained in standard citrated tubes is calculated for a haematocrit of 40%. The lower (25%) and upper (55%) haematocrit limits for using standard citrate tubes, as specified in the recommendation (Kitchen et al., 2021), result in citrate volume errors of ±25% relative to this 40% optimum. Therefore, considering that a deviation of ±25% from the calculated optimum is acceptable, two predefined ranges for haematocrit values of >55% were built, ranges centred on 65% (from 56% to 74%) and 80% (from 75% to 85%) haematocrit, which required 175 and 100 μL of citrate, respectively. In detail, three ranges of haematocrit values were considered for citrate adjustment: for haematocrit of ≤55%, no citrate adjustment was done (citrate volume in the tube, 300 μL); for haematocrit between 56% and 74%, 125 μL citrate was removed to obtain a final amount of 175 μL in the tube; and for haematocrit between 75% and 85%, 200 μL citrate was removed to obtain a final volume of 100 μL in the tube. The excess volume of trisodium citrate was removed from the tube using a tuberculin syringe; this method offers the advantage of preserving the vacuum in the tube (Marlar et al., 2006).

Following standard practices, tourniquet time was minimized; the first volume of blood collected was discarded, and the citrated coagulation tubes were collected first (Kitchen et al., 2021). All tubes used for the analyses were filled properly and mixed gently after collection. Some tubes were used promptly for on‐site analyses (i.e., for ROTEM and complete blood count). From a fraction of the coagulation tubes, platelet‐poor plasma was prepared by double centrifugation (2000*g* for 10 min with intermediate settling) at ambient temperature. The platelet‐poor plasma was then used for haematocrit normalization in ROTEM assays (see section 2.5) or immediately snap‐frozen in liquid nitrogen for storage at −80°C. The frozen samples were subsequently shipped to France in temperature‐controlled packaging with dry ice for thrombin generation assays and for measurement of clotting factors and inhibitors (see section 2.7).

### 2.5 Thromboelastometry at native and normalized haematocrit: assays and parameters

At both altitudes, in LL and highlanders, whole‐blood ROTEM was performed using the same device (ROTEM delta, Werfen, Le‐Pré‐Saint‐Gervais, France) on citrated blood samples collected as described above. Quality control tests were performed weekly (at both altitudes) during the study using the normalized plasma and reagents provided by the ROTEM manufacturer, and all measured values remained within the targeted ranges. All measurements were conducted at 37°C, within 4 h after blood sampling, and all citrated blood tubes were preheated in the dedicated preheating station of the system for 5–10 min before analysis, according to the manufacturer's instructions. For each participant, using the different available channels of the ROTEM, four standard assays (EXTEM, INTEM, FIBTEM and APTEM) were conducted simultaneously (Volod et al., 2022). In the EXTEM test, activation of coagulation with recombinant tissue factor allows exploration of the extrinsic pathway. In the INTEM test, the intrinsic pathway is activated with phospholipids and ellagic acid. With the FIBTEM reagent, activation is identical to EXTEM, but platelets are inhibited by cytochalasin D, to study the relative contributions of fibrinogen and platelets to clot stability. The APTEM reagent is similar to EXTEM but contains aprotinin, allowing, by comparison with EXTEM, the detection hyperfibrinolysis (Volod et al., 2022). For each ROTEM assay, the two most representative parameters were extracted from the generated TEMogram (i.e., the generated curve showing the clot elasticity over time during clot formation and then after lysis): the clotting time (in seconds, representing the time between the activation of coagulation and the detection of a 2‐mm‐amplitude clot) and the clot amplitude at 20 min (in millimetres, corresponding to the clot firmness). The rigorous maximal clot firmness was not always obtained on the TEMogram because of the time constraints of the experiments. However, clot amplitude at 20 min was correlated extremely well with the maximal clot firmness when measured (*R* ^2^ > 0.96, data not shown), in agreement with previous work (Görlinger et al., 2013). In the following, clot amplitude at 20 min is assimilated to the maximal clot firmness. Clotting time, reflecting clot initiation, is affected mainly by clotting factors, whereas maximal clot firmness, reflecting clot strength, is influenced mainly by thrombocytes, fibrinogen, factor XIII and fibrinolysis (Volod et al., 2022). Therefore, an increased clotting time and/or a reduced clot amplitude would be in favour of a hypocoagulable state, whereas a shortened clotting time and/or increased clot amplitude would indicate a hypercoagulable state (Luddington, 2005). Reference ranges at sea level for each parameter were obtained from Lang et al. (2005).

Given that an increase in haematocrit has been shown greatly to decrease clot firmness in ROTEM in normal reconstituted blood (Nagler et al., 2013; Noorman & Hess, 2018; Westbury et al., 2013), it is important to compare the different groups of participants (LL, EE− and EE+) without this haematocrit bias. Therefore, for each highlander participant, the same ROTEM assays were conducted on a second sample, which was normalized to a haematocrit of 40% by haemodilution with autologous platelet‐poor plasma. This was performed for almost all highlander participants (*n* = 42 of 45, 93%), because three ROTEM assays at normalized haematocrit failed for technical reasons.

### 2.6 Haematology assays

#### 2.6.1 Haemoglobin concentration

The [Hb] was measured in duplicate from a venous blood sample (ABL80, Radiometer, Copenhagen, Denmark).

#### 2.6.2 Haematocrit

Haematocrit was measured in duplicate from a venous blood sample, using the microcentrifuge method (Sigma 1‐14, Sigma Laborzentrifugen, Osterode am Harz, Germany).

#### 2.6.3 Platelet count

At low altitude, for LL, the platelet count and mean platelet volume were obtained from a complete blood count performed using a Sysmex XN‐10 analyser (Sysmex, Kobe, Japan). At high altitude, complete blood counts were obtained for all highlanders, after daily car transportation of the EDTA tubes to the closest local laboratory (Puno, Peru) for analysis (BC‐51250 analyser, Mindray, Shenzhen, China), with a maximal delay between blood sampling and analysis of <30 h, a maximal delay acceptable in a remote high‐altitude area (Rocke et al., 2018). The mean storage temperature before transportation was 12 ± 3°C.

### 2.7 Complementary haemostasis assays

All complementary haemostasis assays were performed on deep‐frozen plasma samples (see preparation details section 2.4), previously stored at −80°C until analysis, which were thawed once at 37°C in a water‐bath for 5 min. The panel of haemostasis assays included routine assays [prothrombin time (PT), activated partial thromboplastin time (aPTT), fibrinogen level, clotting factors and inhibitors] and a thrombin generation assay.

#### 2.7.1 Routine haemostasis assays

A STA‐R Max 3 coagulometer (Stago, Asnières, France) was used with the following reagents: STA®‐NeoPTimal (Stago) for PT, STA®‐PTTA (Stago) for aPTT and STA®‐Liquid Fib (Stago) for fibrinogen activity (Clauss method). Prothrombin [factor (F)II], FV and FX levels were determined with STA®‐NeoPTimal and, respectively, with STA®‐Deficient II (Stago), STA®‐Deficient V (Stago) and STA®‐Deficient X (Stago). The FVII level was determined with Dade® Innovin® (Siemens, Marburg, Germany) and with STA®‐Deficient VII (Stago). The FVIII, FIX, FXI and FXII levels were determined with STA®‐CK Prest® (Stago) and, respectively, with STA®‐Immunodef VIII (Stago), STA®‐Immunodef IX (Stago), STA®‐Immunodef XI (Stago) and STA®‐Imminodef XII (Stago). Antithrombin activity (AT), protein C activity (PC) and free protein S antigen (PS) were determined, respectively, with STA®‐Stachrom® Antithrombin (Stago), STA®‐Stachrom® Protein C activity and STA®‐ Liatest® Free Protein S (Stago). D‐Dimers were determined with STA®‐Liatest D‐Di (Stago). Photometric determination of FXIII activity was done using the Berichrom® Factor XIII assay (Siemens).

To consider the ‘functional’ fibrinogen concentration according to the potential dilution effect of red blood cells in highlanders, a whole‐blood fibrinogen concentration was calculated using the following formula: whole‐blood fibrinogen concentration (in grams per litre) = (1 − haematocrit) × plasma fibrinogen concentration (in grams per litre), as previously described (Rupa‐Matysek et al., 2016; Solomon et al., 2012; Westbury et al., 2013).

#### 2.7.2 Thrombin generation

A calibrated automated thrombogram assay (Thrombinoscope BV, Maastricht, Netherlands) on an automated fluorometer (Fluoroscan Ascent, ThermoLab Systems, Franklin, MA, USA) was performed according to the manufacturer's instructions and using the manufacturer's software. Coagulation was triggered by 5 pM of tissue factor and 4 μM of phospholipids [PPP Reagent® (Thrombinoscope BV)]. All thrombin generation assays were run in triplicate in 96‐well plates in standard conditions. Briefly, 80 μL of PPP Reagent® was mixed with 20 μL of tissue factor/phospholipids. Plates were incubated for 10 min at 37°C in the automated fluorimeter before adding the fluorogenic substrate (ZGGR‐AMC) and calcium (FluCa Kit®, Stago, France). Raw data were analysed using the software Thrombinoscope v.5.0 (Thrombinoscope BV). The lag time (time to start of thrombin generation, in minutes), time to peak (in minutes), peak height (nanomolar) and endogenous thrombin potential (nanomolar per minute) were recorded (Tripodi, 2020). Owing to the reduced amount of platelet‐poor plasma available (because of the very high haematocrits), the thrombin generation assay was not performed in 2 of 15 (13%) EE− and 13 of 30 (43%) EE+, and not performed in 1 of 10 (10%) LL, owing to technical failure.

### 2.8 Statistical analysis

Owing to the exploratory nature of this observational study, no sample size estimation was performed. Categorical data (sex, EE and CMS status) were expressed as absolute counts and percentages. Continuous data (physiological, haematological and haemostasis variables) were expressed as the mean ± SD or median [25th–75th percentiles], depending on whether the data were normally distributed or not. One‐way ANOVA or Kruskal–Wallis tests were conducted to assess differences between the three groups of participants (control LL, EE− and EE+); in the event of a significant main effect, *post hoc* pairwise comparisons were performed using Tukey's HSD test or Dunn's test with Bonferroni correction, as appropriate. Given that many ROTEM parameters exhibited skewed distributions, all were expressed and analysed as non‐parametric variables, and no covariate adjustment was performed. Two statistical analyses were conducted on the ROTEM data: first, the test results were compared at native haematocrit between LL, EE− and EE+; and second, the results obtained on samples at normalized haematocrit in EE− and EE+ were compared with LL results (conducted only at native haematocrit, given that all LL haematocrits were <50%). In addition, to explore the difference induced by the haemodilution procedure in EE+, ROTEM results obtained from samples without and with haemodilution were compared using Wilcoxon's signed‐rank test. Exploratory association analyses were conducted with correlation analysis using Spearman's rank correlation coefficients (ρ) or bivariate linear regression (*R* ^2^), when appropriate.

All tests were two sided, and a *P*‐value of <0.05 was considered significant. All statistical analyses were performed using the software R (v.4.2.2, The R Foundation for Statistical Computing, Vienna, Austria) and GraphPad Prism (v.9.5.0, GraphPad Software, Boston, MA, USA).

## 3 RESULTS

### 3.1 General characteristics

**FIGURE 1:**
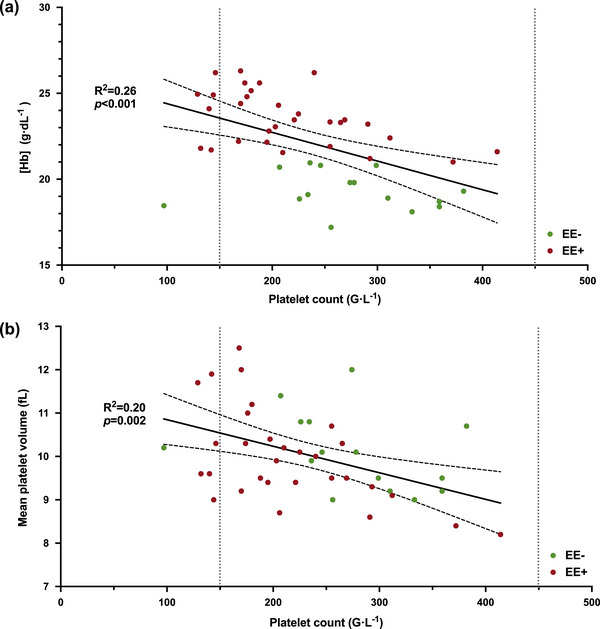
Platelet count in highlander participants permanently living at 5100 m a.s.l., without (EE−) and with (EE+) excessive erythrocytosis, presented an inverse linear relationship with the haemoglobin concentration ([Hb]) (a) and the mean platelet volume (b). Normal ranges (lower and upper limit values) for each parameter are represented by the dotted lines.

The demographic characteristics of the 55 participants are summarized in Table 1. Almost all EE+ suffered from CMS, mainly of mild severity. As expected, EE+ presented the highest [Hb] and haematocrit values among the groups, whereas the values of the EE− group were intermediate between those of the EE+ and LL groups. The EE+ also exhibited significantly lower platelet counts than EE− (Table 1), albeit mostly within the normal range of clinical values (24 of 30, 80%; Figure 1). In highlander participants, platelet count showed a negative linear relationship with [Hb] (*R* ^2 ^= 0.26, *P *< 0.001; Figure 1a) and a negative relationship with mean platelet volume (*R* ^2 ^= 0.20, *P *= 0.002; Figure 1b).

**TABLE 1:** Demographic and haematological characteristics of the participants and conventional coagulation tests.

Variables	LL, 210 m a.s.l. (*n* = 10)	EE−, 5100 m a.s.l. (*n* = 15)	EE+, 5100 m a.s.l. (*n* = 30)	*P*‐value
Male sex	8 (80%)	15 (100%)	30 (100%)	_
Age (years)	33 ± 7	40 ± 13	48 ± 9^*^	<0.001
Body mass index (kg m^−2^)	22.4 ± 2.3	26.0 ± 3.3^*^	27.2 ± 3.5^*^	0.001
SpO_2_ (%)	97 ± 1	83 ± 4^*^	82 ± 5^*^	<0.001
CMS status				
No CMS	_	15 (100%)	2 (7%)	_
Mild CMS	_	0 (0%)	19 (63%)	
Moderate‐to‐severe CMS	_	0 (0%)	9 (30%)	
[Hb] (g dL^−1^)	14.7 ± 1.2	19.3 ± 1.1^*^	23.5 ± 1.6^*^, ^†^	<0.001
Haematocrit (%)	43 ± 4	56 ± 3^*^	70 ± 6^*^, ^†^	<0.001
Platelet count (×10^9^ L^−1^)	235 ± 28	273 ± 73	216 ± 70^†^	0.03
Mean platelet volume (fL)	10.1 ± 0.8	10.1 ± 0.9	10.0 ± 1.1	0.92
Fibrinogen (g L^−1^)				
Plasma fibrinogen	2.6 ± 0.2	3.0 ± 0.4	3.5 ± 0.8^*^	0.001
Whole‐blood fibrinogen concentration	1.4 ± 0.1	1.3 ± 0.2	1.0 ± 0.3^*^, ^†^	<0.001
PT (s)	13.7 [13.1–13.9]	13.2 [12.5–14.2]	13.4 [13.0–14.4]	0.95
aPTT (s)	34.0 ± 2.9	36.9 ± 5.3	36.0 ± 4.2	0.27
D‐dimers (mg L^−1^)	0.27 [0.27–0.29]	0.27 [0.27–0.34]	0.31 [0.27–0.42]	0.07

*Note*: Categorical data are reported as the absolute count and percentage (%). Continuous data are reported as the mean ± SD or median [25th–75th percentiles].

Abbreviations: aPTT, partial thromboplastin time; CMS, chronic mountain sickness; EE−, highlanders without excessive erythrocytosis; EE+, highlanders with excessive erythrocytosis; [Hb], haemoglobin concentration; LL, lowlanders; PT, prothrombin time; SpO_2_, oxygen saturation assessed by finger pulse oximetry.

* *P* < 0.05 EE+ or EE− vs. LL.

^†^ *P* < 0.05 EE+ vs. EE−.

Fibrinogen levels in EE+ were significantly higher than those in EE− and LL; interestingly, when considering the whole‐blood fibrinogen concentration, the EE+ group exhibited the lowest fibrinogen values among the groups (Table 1). No statistically significant differences were observed among the three groups regarding first‐line haemostasis assays (i.e., aPTT, PT and D‐dimers; Table 1). According to the dedicated questionnaire, no history of confirmed venous thromboembolism was identified in highlander participants.

### 3.2 Clotting factors and inhibitors

**FIGURE 2:**
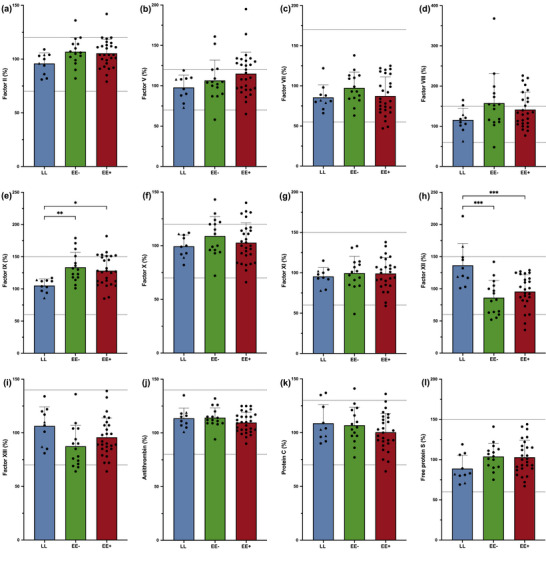
Plasma levels of clotting factors and inhibitors are similar among the lowlander (LL, 210 m a.s.l.), highlander without (EE−) and with excessive erythrocytosis (EE+) groups (5100 m a.s.l.). The bar graphs represent mean values with SD in each group, with individual values (black dots for men, and black triangles for women only in the LL group). Normal ranges (lower and upper limit values) for each parameter are represented by the dotted lines. ^*^ *P *< 0.05, ^**^ *P *< 0.01 and ^***^ *P *< 0.001. Individual missing data, only in the EE+ group: factor II (*n* = 3), factor V (*n* = 2), factor VII (*n* = 3), factor VIII (*n* = 4), factor IX (*n* = 3), factor X (*n* = 1), factor XI (*n* = 4), factor XII (*n* = 5), factor XIII (*n* = 3), antithrombin (*n* = 1), protein C (*n* = 3) and free protein S (*n* = 3).

Plasma levels of clotting factors and inhibitors, with individual values, are presented in Figure 2. Only FIX (Figure 2e) and FXII (Figure 2h) were significantly higher and lower, respectively, in both highlander groups than in LL. However, the mean values of all the factors and inhibitors were within or close to the normal range (Figure 2a–l), indicating normal plasma coagulation.

### 3.3 Rotational thromboelastometry measurements

**FIGURE 3:**
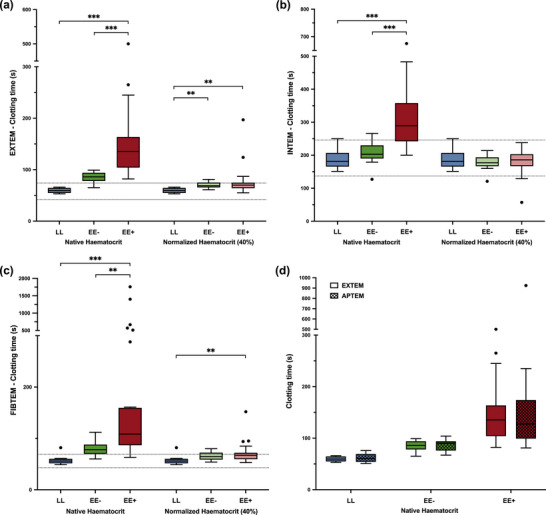
At native haematocrit, highlanders permanently living at 5100 m a.s.l. with excessive erythrocytosis (EE+) exhibited a large increase in clotting time compared with highlanders without excessive erythrocytosis (EE−) and lowlanders (LL) at 210 m a.s.l., which was almost totally corrected when ROTEM tests were secondarily performed at normalized haematocrit (40%) after haemodilution with autologous platelet‐poor plasma. (a–c) Clotting time in EXTEM (a), INTEM (b) and FIBTEM (c) assays. (d) Comparisons between APTEM and EXTEM clotting times at native haematocrit in each subgroup of participants. Boxplots represent the median with 25th and 75th percentiles (lower and upper hinges). Whiskers extend from the corresponding hinge to the highest or lowest value not further than 1.5 × interquartile range. Horizontal dashed lines represent the reference ranges established at sea level (Lang et al., 2005). ^**^ *P *< 0.01 and ^***^ *P *< 0.001.

**FIGURE 4:**
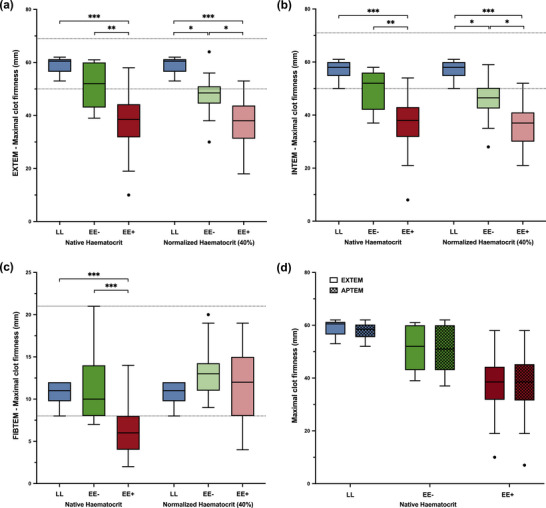
At native haematocrit, highlanders permanently living at 5100 m a.s.l. with excessive erythrocytosis (EE+) exhibited a large reduction in maximal clot firmness compared with highlanders without excessive erythrocytosis (EE−) and lowlanders (LL) at 210 m a.s.l., which was not corrected, except in the FIBTEM assay, when ROTEM assays were secondarily performed at normalized haematocrit (40%) after haemodilution with autologous platelet‐poor plasma. (a–c) Maximal clot firmness in EXTEM (a), INTEM (b) and FIBTEM (c) assays. (d) Comparisons between APTEM and EXTEM maximal clot firmness at native haematocrit in each subgroup of participants. Boxplots represent the median with 25th and 75th percentiles (lower and upper hinges). Whiskers extend from the corresponding hinge to the highest or lowest value not further than 1.5 × interquartile range. Horizontal dashed lines represent the reference ranges established at sea level (Lang et al., 2005). ^*^ *P *< 0.05, ^**^ *P *< 0.01 and ^***^ *P *< 0.001.

Results from the ROTEM assays are depicted in Figures 3 and 4. At native haematocrit, the EE+ group exhibited a hypocoagulable profile compared with the EE− and LL groups. First, there was a delayed clot initiation, with a significant increase in clotting time in all ROTEM assays (Figure 3a–c). Second, EE+ showed a much lower maximal clot firmness than both EE− and LL in all ROTEM assays (Figure 4a–c). Comparisons between the EXTEM and APTEM assays did not support hyperfibrinolysis among the groups (Figures 3d and 4d).

Haematocrit normalization of highlander samples to a target haematocrit of 40% was successful, because the post‐dilution haematocrit measurements gave an average value of 40 ± 4%. The ROTEM profiles obtained from those haematocrit‐normalized samples were partly modified in all assays with respect to those obtained at native haematocrit (Figures 3 and 4). First, the haematocrit normalization eliminated the differences in clotting time between the EE+ and EE− groups in all assays (Figure 3a–c). Although clotting times were still slightly higher in the normalized highlander groups than in the LL group in EXTEM and FIBTEM (Figure 3a,c), most values were within the normal range. Unexpectedly, haematocrit normalization eliminated significant differences in maximal clot firmness between groups only in the FIBTEM assay (Figure 4c), whereas in both EXTEM (*P *= 0.66) and INTEM (*P *= 0.63) assays, the EE+ maximal clot firmness was not modified (Figure 4a,b).

**FIGURE 5:**
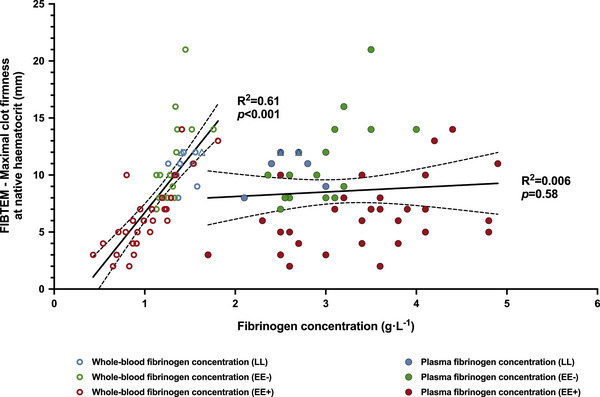
At native haematocrit, in the overall population, only whole‐blood fibrinogen concentration, and not plasma concentration, showed a significant linear relationship with maximal clot firmness in FIBTEM assay. Abbreviations: EE−, highlanders without excessive erythrocytosis; EE+, highlanders with excessive erythrocytosis; LL, lowlanders. Triangles represent individual values for women (LL group only).

Maximal clot firmness in EXTEM and INTEM was moderately correlated with platelet count (ρ = 0.50 and ρ = 0.52, respectively, *P *< 0.001) and strongly correlated with whole‐blood fibrinogen (ρ = 0.85 and ρ = 0.84, respectively, *P *< 0.001), but not with plasma fibrinogen concentration (*P *> 0.05). Regarding the association between clot strength in FIBTEM and fibrinogen concentration, FIBTEM maximal clot firmness showed a significant linear relationship with whole‐blood fibrinogen (*R* ^2 ^= 0.61, *P *< 0.001), but not with plasma concentration (Figure 5).

### 3.4 Thrombin generation assay

**FIGURE 6:**
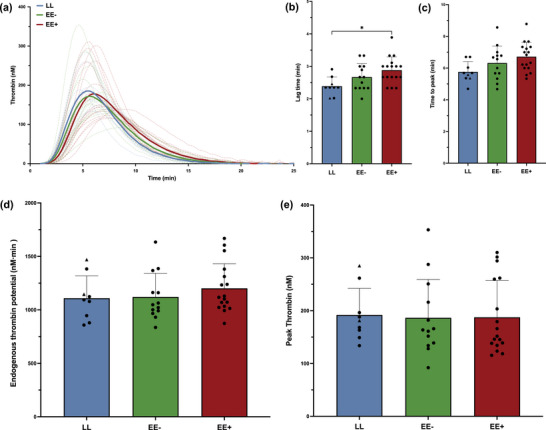
Thrombin generation patterns are similar among the groups, with a significant longer lag time only in highlanders with excessive erythrocytosis (EE+; 5100 m a.s.l.) versus lowlanders (LL; 210 m a.s.l.). Abbreviations: EE−, highlanders without excessive erythrocytosis; EE+, highlanders with excessive erythrocytosis; LL, lowlanders. (a) Visual reconstruction of individual thrombin generation curves (dashed lines), with mean curves (thick lines). The bar graphs represent the mean values with SD in each group, with individual values (black dots for men, and black triangles for women only in the LL group) for lag time (b), time to peak (c), endogenous thrombin potential (d) and peak thrombin (e). ^*^ *P *< 0.05.

The thrombin generation assay results are shown in Figure 6. No significant differences were found between the groups in the time to peak (Figure 6c), the endogenous thrombin potential (Figure 6d) and the peak thrombin (Figure 6e). The only change concerned the lag time, which was slightly increased in EE+ versus LL (*P *= 0.01; Figure 6b).

## 4 DISCUSSION

In this observational study, using a haemostasis testing panel that included ROTEM assays conducted in the field at 5100 m a.s.l., we determined the whole‐blood and plasma coagulation patterns of high‐altitude native highlanders living permanently at >5000 m a.s.l., focusing on those suffering from maladaptation (i.e., EE) compared with both a group of highlanders without EE and a seal‐level control group of lowlanders. To our knowledge, this is the first study using ROTEM to evaluate the whole‐blood coagulation status of highlanders with and without EE. The main findings of this study were as follows: first, EE+ exhibited a strong hypocoagulable ROTEM profile compared with both EE− and LL, partly corrected after haemodilution with platelet‐poor plasma; second, EE+ and EE− had a normal plasma coagulation, very similar to that of LL; and third, EE− exhibited similar ROTEM and plasma coagulation patterns to LL. Taken together, in contrast to our hypothesis, these results do not suggest a hypercoagulable profile in Andean highlanders with EE living permanently at extreme altitudes.

The main result of our cross‐sectional study is the apparent hypocoagulable ROTEM profile observed in EE+ at native haematocrit compared with EE− and LL. This profile is characterized by a considerable delay in clot initiation and much lower maximal clot firmness in all reagent assays. The similar amount of generated thrombin and clotting factor levels in EE+ compared with the other groups (Figures 2 and 6) does not support the hypothesis of a thrombin generation deficiency to explain any of those differences. The usual haemostatic assays (i.e., PT and aPTT; Table 1) and plasma levels of clotting factors and inhibitors (Figure 2) were mostly not affected either by permanent life in hypoxia (i.e., in EE−) or by EE. In agreement with the present study, DeSouza et al. (2021) reported no difference in FVII, FVIII and FX levels between EE+ and EE− residing ∼1000 m lower, in Cerro de Pasco (Peru). Studies conducted on the Qhingai‐Tibet plateau reported conflicting results regarding differences in aPTT and PT values between EE/CMS and control highlanders (Jiang et al., 2021; Yin et al., 2022). However, these results should be interpreted with caution, because no citrate‐to‐blood ratio adjustment for EE+ is mentioned and because they were performed in different ethnic groups.

The very high haematocrits observed in EE+ suggest clinical proximity to patients affected by polycythaemia vera. In this context, Tripodi et al. (2013) reported lower endogenous thrombin potential in thrombin generation tests (using platelet‐poor plasma) and a similar ROTEM profile in the INTEM assay compared with healthy control subjects. Conversely, others reported a hypercoagulable ROTEM profile in polycythaemia vera (Şahin et al., 2021); however, those patients with polycythaemia vera exhibited lower haematocrit or [Hb] levels than those in the present study, and factors other than haematocrit are implicated in the prothrombotic tendency associated with polycythaemia vera (Gangaraju et al., 2020; Kroll et al., 2015). The very high haematocrits and hypocoagulable ROTEM profile observed in EE+ subgroup were qualitatively similar to those previously reported in chronically hypoxaemic patients at sea level suffering from uncorrected cyanotic congenital heart disease (CCHD) (Pujol et al., 2019; Rupa‐Matysek et al., 2016; Westbury et al., 2013). Similar to the EE+ participants, CCHD patients had significantly prolonged clotting times and higher plasma fibrinogen concentrations than control subjects, but lower whole‐blood fibrinogen concentration (Westbury et al., 2013). They also exhibited notably lower maximal clot firmness in all ROTEM assays (Pujol et al., 2019; Rupa‐Matysek et al., 2016; Westbury et al., 2013). Quantitatively, however, in EXTEM, the difference in maximal clot firmness was considerably larger between EE+ and LL (median of 22 mm; Figure 4a) than previously reported between CCHD patients and control subjects (13 mm) (Westbury et al., 2013). Also, in most CCHD studies, CCHD patients showed severe thrombocytopenia (i.e., platelet count <100 × 10^9^ L^−1^) (Lill et al., 2006; Westbury et al., 2013), which is not the case in our study (Table 1; Figure 1).

Westbury et al. (2013) hypothesized that prolonged clotting times and lower FIBTEM and EXTEM clot firmness in CCHD patients with high haematocrits were caused by the relative dilution of fibrinogen and clotting factors by the increased relative volume of red cells; this hypothesis was validated quantitatively in the same study by performing EXTEM and FIBTEM assays on a high‐haematocrit blood model. A significant part of our data can be explained by this model because, after haematocrit normalization at 40% using un‐manipulated platelet‐poor plasma, both FIBTEM clotting time and maximal clot firmness in EE+ were normalized to EE− and LL levels (Figure 4c). Moreover, the long clotting times in EE+ in all assays at native haematocrit were also almost corrected by haematocrit normalization (Figure 3a–c). Thus, the large increases in clotting times in all assays and the difference in maximal clot firmness in FIBTEM can be explained by Westbury's dilution model. Nevertheless, a significant portion of our data could not be explained by Westbury's dilution model. The EE+ EXTEM and INTEM maximal clot firmness remained unchanged after dilution (*P *= 0.66, EXTEM; *P *= 0.63, INTEM) and very small (−22 mm vs. LL; Figure 4a,b). By design (i.e., the use of platelet‐poor plasma), the normalized EE+ dilution factor was the same for all haematological parameters, except for platelets. Platelet dilution might then be responsible for the absence of changes in EXTEM and INTEM maximal clot firmness. However, as shown in previous experimental reports, the maximal clot firmness exhibits a biphasic behaviour as a function of platelet concentration, which first increases sharply with platelet concentration up to 100 × 10^9^ L^−1^, then remains almost constant (Bontekoe et al., 2019; Lang et al., 2009; Maslow et al., 2023; Noorman & Hess, 2018). In the EE+ subgroup, the haematocrit normalization should have produced an estimated platelet concentration of 131 ± 51 × 10^9^ L^−1^, ranging from 65 × 10^9^ to 285 × 10^9^ L^−1^. According to Bontekoe et al. (2019) and Maslow et al. (2023), platelet concentrations of <60 × 10^9^ L^−1^ would be needed to predict the large decrease in maximal clot firmness observed between EE+ and LL at normalized haematocrit in both EXTEM and INTEM assays (Figure 4a,b). Recalling that the FIBTEM assay differs from the EXTEM assay only in the inhibition of platelets by cytochalasin D, it follows that the absence of normalization of the INTEM assay with respect to the FIBTEM assay suggests an abnormal behaviour of the platelets (e.g., decrease in platelet contribution to clot strength and/or abnormal platelet–fibrinogen interaction and/or abnormal platelet–red cell interaction; Weisel & Litvinov, 2019). However, given that control of the exact platelet count on the haemodiluted samples was not possible (owing to the logistic constraints and the absence of biological facilities in this highly remote city), no further conclusions can be drawn regarding the platelet contribution to clot strength.

In order to provide a relatively exhaustive overview of the process of haemostasis in highlanders, this study lacks a specific assessment of the fibrinolysis phase and of primary haemostasis. Our data (including APTEM assays results and dosages of D‐dimers) did not support a hyperfibrinolytic state in EE+, which is consistent with previous observations (DeSouza et al., 2021; Jiang et al., 2021). The trend to higher levels of D‐dimers in the EE+ group might be explained by the older age of the EE+ participants. Regarding primary haemostasis, we observed a decrease in platelet count between EE− and EE+ similar to those previously reported in EE/CMS highlanders, for which different underlying mechanisms have been proposed (Wang et al., 2022, 2023) Among them, the inverse relationship between platelet count and mean platelet volume (Figure 1b) might suggest that an increased peripheral consumption rather than reduced platelet production might explain the lower platelet count in EE+, because newly formed platelets are known to have larger volume. This might be in agreement with a recent study suggesting an increased platelet activation and thus apoptosis in EE+, where a similar negative correlation between platelet count and mean platelet volume, in addition to lower platelet count, were shown in EE+ (Wang et al., 2023).

The highlanders included in the present study did not report any history of venous thromboembolism based on the dedicated survey (Frezzato et al., 1996). This finding could support that an adaptive qualitative whole‐blood coagulation process involving platelets might counterbalance the potential prothrombotic effect attributed to high haematocrits in EE+ (Ortiz‐Prado et al., 2022; Villafuerte & Corante, 2016; Villafuerte et al., 2022). Nevertheless, the small sample size of our study does not allow us to draw epidemiological conclusions, and in addition to physiological investigations, large clinical and epidemiological studies are mandatory to assess specifically the incidence of venous or arterial thrombotic disorders in highlander populations.

Finally, our results provide important insight regarding the conflicting results reported in two recent studies on Andean highlanders. Our results agree with the coagulation–fibrinolytic investigation conducted in Cerro de Pasco (∼4300 m a.s.l., Peru), which did not support a hypercoagulable pattern in EE+ (DeSouza et al., 2021). Conversely, the present results apparently contradict the preliminary study that we conducted in La Rinconada (5100 m a.s.l.), in which bleeding and clotting times were measured in EE+ and compared with those of EE− (Hancco, Champigneulle, et al., 2020). Given that low haematocrits are a known confounding factor in the PFA‐100® system, which is the modern version of bleeding time measurements, one could speculate that very high haematocrits in EE+ should have the opposite effect (i.e., a shortening of bleeding times), as observed in our preliminary study (Lordkipanidzé, 2016; Small et al., 1983).

Our study presents some limitations that should be acknowledged. First, the ROTEM allows a global evaluation of the clotting functions, but a detailed investigation of platelet qualitative dysfunctions requires specific modifications of the assay or other specific assays (e.g., light transmission aggregometry, whole‐blood aggregometry or flow cytometry analysis) that were not performed here (Lordkipanidzé, 2016; Racine‐Brzostek & Asmis, 2020; Solomon et al., 2015; Volod et al., 2022). Also, the ROTEM assays measure clot formation at low shear rates (0.1 s^−1^), as in venous system (Lang et al., 2009), and does not reflect the intra‐arterial clot formation mechanisms, mainly involving platelets, subendothelial collagen and von Willebrand factor (Volod et al., 2022; Weisel & Litvinov, 2019). Finally, as previously done (Stauffer et al., 2020), the LL sea‐level control group included in our study consisted of healthy European participants and not Peruvian lowlanders, leading to an LL group that was not matched with the highlander groups for age, ethnicity and sex. Nevertheless, ROTEM profiles have been shown to be only marginally influenced by age and sex, with a slight procoagulant trend in ROTEM pattern usually observed with ageing (Lang et al., 2005). Therefore, the age difference between groups in our study might have induced an underestimation of the hypocoagulable profile of EE+ compared with LL.

**FIGURE 7:**
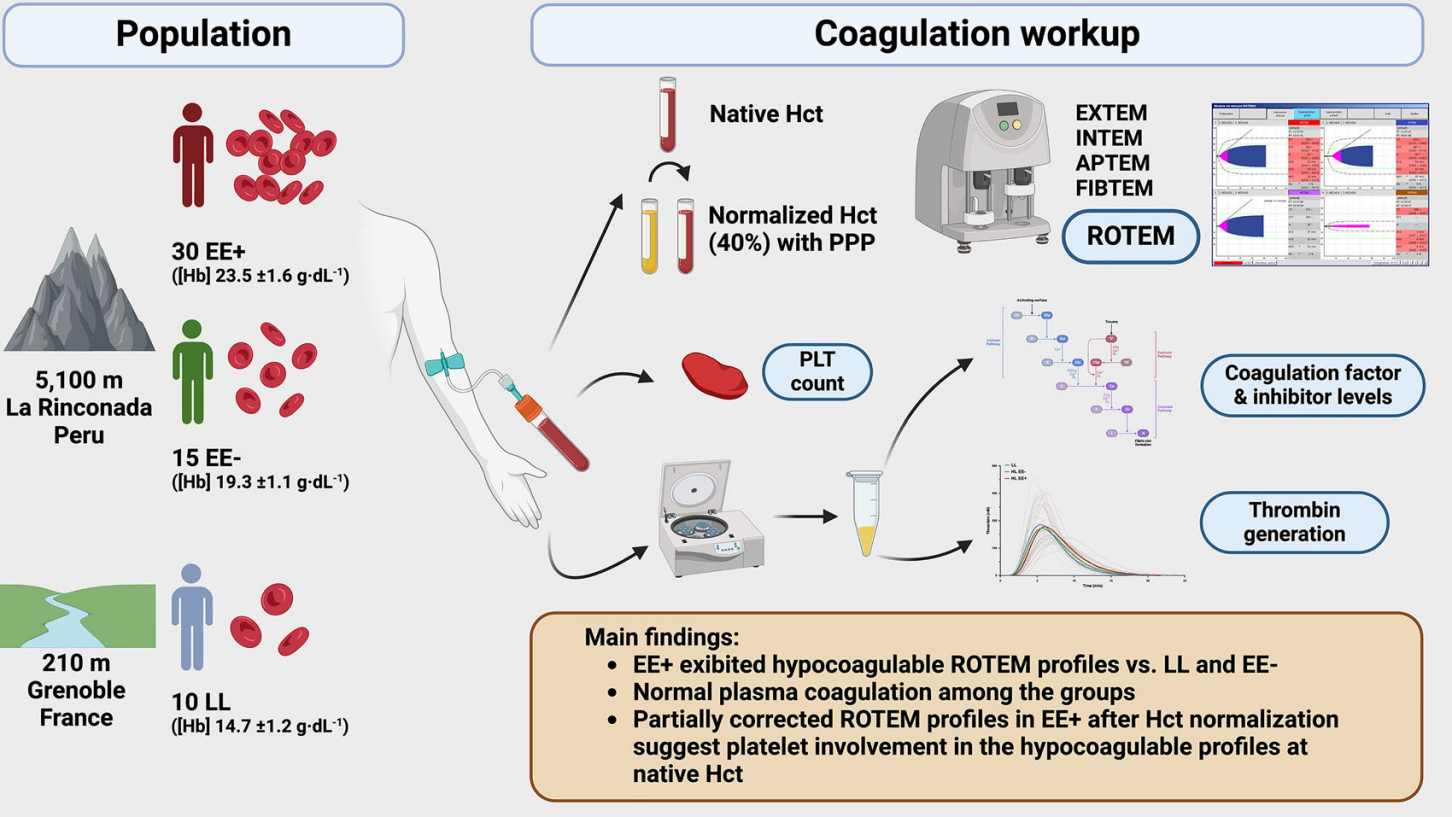
Summary of the coagulation work‐up performed and main conclusions regarding coagulation pattern in highlanders with (EE+) and without (EE−) excessive erythrocytosis living permanently above 5000 m a.s.l. in the highest city in the world (La Rinconada, Peru, 5100–5300 m a.s.l.). Abbreviations: EE+, highlanders with excessive erythrocytosis; EE−, highlanders without excessive erythrocytosis; [Hb], haemoglobin concentration; Hct, haematocrit; LL, lowlanders; PLT, platelet; PPP, platelet‐poor plasma; ROTEM, rotational thromboelastometry.

In conclusion, we observed in this cross‐sectional study conducted at extreme altitude, that EE was not associated with a hypercoagulable state. Furthermore, compared with both healthy LL and EE−, EE+ exhibited a hypocoagulable ROTEM profile that could not be reduced to a simple red blood cell dilution effect as observed in CCHD. The absence of maximal clot firmness normalization after haematocrit normalization in EXTEM/INTEM, together with the FIBTEM normalization, suggests that platelets might play a special role through either genetic or dietary adaptations. These main and major findings, in addition to the coagulation work‐up performed in the study, are summarized in the Figure 7. Further studies are necessary to understand the haemostatic process in native Andean highlanders, potentially impacted both by chronic hypoxia exposure and polycythaemia, and to determine whether the present findings might represent an adaptive benefit to high‐altitude life. Moreover, well‐conducted large‐scale epidemiological studies are mandatory to assess specifically the incidence of both venous and arterial thrombotic events in the Andean highlander population, independently of the common associated risk factors.

## AUTHOR CONTRIBUTIONS

Benoit Champigneulle, François Caton, Émeric Stauffer, Aurélien Pichon, Julien V. Brugniaux, Paul Robach, Philippe Connes, Pierre Bouzat, Benoit Polack, Raphael Marlu and Samuel Verges designed the study. Benoit Champigneulle, Landry Seyve, Émeric Stauffer, Aurélien Pichon, Julien V. Brugniaux, Michael Furian, Ivan Hancco, Blandine Deschamps and Lars Kaestner performed the experimentations and/or acquired the data. Benoit Champigneulle performed the statistical analysis. Benoit Champigneulle, François Caton, Landry Seyve, Benoit Polack, Raphael Marlu and Samuel Verges analysed and interpreted the data. Benoit Champigneulle, François Caton and Samuel Verges wrote the manuscript; Landry Seyve, Émeric Stauffer, Aurélien Pichon, Julien V. Brugniaux, Michael Furian, Ivan Hancco, Blandine Deschamps, Lars Kaestner, Paul Robach, Philippe Connes, Pierre Bouzat, Benoit Polack and Raphael Marlu revised it critically for important intellectual content. All authors approved the final version of the manuscript and agree to be accountable for all aspects of the work in ensuring that questions related to the accuracy or integrity of any part of the work are appropriately investigated and resolved. All persons designated as authors qualify for authorship, and all those who qualify for authorship are listed.

## CONFLICT OF INTEREST

The authors declare no conflicts of interest.

## Data Availability

The datasets supporting the findings of the present study are available from the corresponding author on reasonable request.
